# Ischaemic Stroke as a Manifestation of Marantic Endocarditis in Lung Adenocarcinoma

**DOI:** 10.7759/cureus.112034

**Published:** 2026-07-04

**Authors:** Elisa Caldeira, Carolina Barros, Patrício Freitas

**Affiliations:** 1 Internal Medicine, Hospital Central do Funchal, Funchal, PRT; 2 Stroke Unit, Hospital Central do Funchal, Funchal, PRT

**Keywords:** embolism, lung adenocarcinoma, marantic endocarditis, stroke, therapeutic anticoagulation

## Abstract

Marantic endocarditis (ME) is characterised by the formation of sterile valvular vegetations, carrying a high risk of systemic embolism. It is frequently associated with advanced neoplasms, particularly adenocarcinomas of the lung. Osimertinib, often used in lung adenocarcinomas, is linked with thrombocytopenia. We report the case of a 57-year-old female with stage IV lung adenocarcinoma on osimertinib, who presented with acute neurological deficits due to an M2 segment occlusion of the left middle cerebral artery, which was treated with mechanical thrombectomy. The post-procedure course was marked by haemorrhagic transformation and systemic embolic events, including splenic ischaemia and the appearance of Janeway lesions. Transthoracic echocardiography identified mitral valve vegetations which, in the context of negative blood cultures and multiple embolic phenomena, made the picture of ME highly suggestive. Due to significant haemorrhagic risk, given the severe thrombocytopenia and haemorrhagic transformation, therapeutic anticoagulation was not feasible. The patient’s clinical state deteriorated rapidly following deep vein thrombosis and pulmonary embolism, culminating in multiorgan failure and death. In this complex case, therapeutic strategy was limited by important thrombocytopenia and the haemorrhagic risk associated with stroke, precluding anticoagulation - a cornerstone of treatment for ME -and thereby affecting the patient’s prognosis.

## Introduction

Non-bacterial thrombotic endocarditis, or marantic endocarditis (ME), is a rare condition characterised by the formation of sterile valvular vegetations that can subsequently embolise to various organs, with strokes being the most common clinical presentation, occurring in 50-60% of cases [1-4]. Diagnosis is challenging as it often mimics infective endocarditis (IE), so echocardiography, negative blood cultures, and evidence of systemic embolism are crucial [1,2]. Furthermore, it is almost always detected too late, since clinical symptoms usually do not appear until after embolic complications have occurred, and this delay in diagnosis has prognostic implications, with high hospital mortality rates [4]. It is frequently associated with advanced neoplasms, particularly adenocarcinomas of the lung, pancreas and gastrointestinal tract, and its treatment consists of early anticoagulation to reduce thromboembolism and treatment of the underlying neoplasm to reduce the prothrombotic stimulus [1,2]. This link is related to a state of systemic hypercoagulability that the neoplasms themselves induce through the release of procoagulant factors (such as tissue factor) and inflammatory cytokines [2,4]. These mechanisms, together with damage to the valvular endothelium, promote the continuous deposition of fibrin and platelets, resulting in the formation of sterile vegetations [1]. This case highlights a therapeutic dilemma in a cancer patient taking osimertinib who developed thrombocytopenia and suffered an ischaemic stroke caused by ME, with haemorrhagic transformation, making it difficult to implement the recommended treatment and emphasising the need for a holistic, multidisciplinary approach.

## Case presentation

A 57-year-old patient with a personal history of stage IV EGFR (epidermal growth factor receptor)-positive lung adenocarcinoma, currently being treated with osimertinib for five years, with no personal history of thromboembolic events in the past, presented to the emergency department with speech impairment and right upper limb weakness, with five hours of evolution. On physical examination, the patient was alert and oriented. Cardiopulmonary auscultation revealed no audible murmurs. Neurological examination showed dysarthria, aphasia, right central facial paresis, and right upper limb paresis, scoring 6 on the NIHSS (National Institutes of Health Stroke Scale) [5]. The patient underwent a computed tomography (CT) scan with an angiographic study, which revealed an acute ischaemic injury secondary to an occlusion of the M2 segment of the left middle cerebral artery. The case was discussed with the interventional neuroradiology team, and mechanical thrombectomy was performed without immediate complications. Subsequently, the patient was admitted to the Stroke Unit. Laboratory findings revealed thrombocytopenia, anaemia, and a new-onset acute kidney injury, with elevated troponin T (Table 1).

**Table 1:** Laboratory results at the time of admission (days 2, 4, 10, and 23 of hospitalisation) Throughout the hospital stay, there was no improvement in laboratory findings; on the contrary, thrombocytopenia persisted, and renal function and markers of myocardial necrosis worsened, reflecting the onset of multiple organ failure, which ultimately led to the patient’s death on day 25. Regarding thrombocytopenia, the patient’s platelet count had been between 92,000 and 110,000 in the three months prior to the onset of symptoms and had worsened by the time of admission. WBC - White Blood Count; MCV - Mean Corpuscular Volume; CRP - C-Reactive Protein; GFR - Glomerular Filtration Rate; CK - Creatine Kinase.

Parameter (reference values)	Admission	Day 2	Day 4	Day 10	Day 23
WBC (×10³/µL) (4.2–10.8)	8.8	13.0	13.2	12.0	12.9
Neutrophils (×10³/µL) (1.9–7.2)	7.7	9.9	9.4	8.8	9.2
Haemoglobin (g/dL) (11.9–14.9)	10.9	11.1	10.8	8.5	8
MCV (fL) (80–99)	89	88.9	87.8	86.5	86.8
Platelets (×10³/µL) (144–444)	61	43	39	35	45
CRP (mg/dL) (<5.0)	5.25	50	94	143	64
Creatinine (mg/dL) (0.5–0.9)	1.48	1.17	1.19	1.07	5.01
GFR (mL/min/1.73 m^2^) (≥60)	39	52	51	58	9
Troponin Ths (ng/mL) (<0.014)	0.376	-	-	0.144	2.670
CK (U/L) (<170)	210	154	185	35	539

After 24 hours, the patient’s neurological status deteriorated, with right hemiparesis, worsening aphasia, and right homonymous hemianopia, leading to an increase in the NIHSS score to 12 [5]. An immediate CT scan was performed, which demonstrated haemorrhagic transformation (Figure 1).

**Figure 1:**
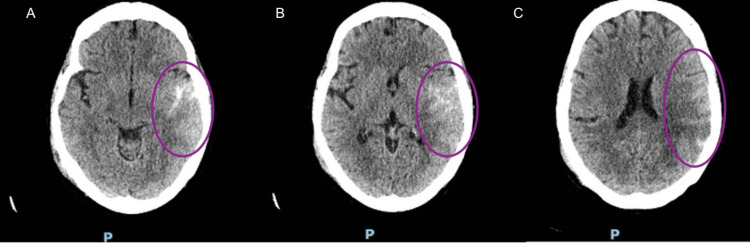
CT scan performed after 24 hours A, B, and C show different levels of area of cortico-subcortical dedifferentiation in the left post-central/parietal region, consistent with a progressive ischaemic lesion in the territory of the left middle cerebral artery, distal branches. This is associated with foci of perisylvian and ipsilateral insular subarachnoid haemorrhage consistent with haemorrhagic transformation (the whitest areas within the brain, inside the purple circle). This area of interest is inside the purple circle.

On the second day of admission, scattered blue-purple macules were observed on the fingers and toes, soles, and palms; these were painless and had a heterogeneous distribution, consistent with Janeway lesions (Figure 2). Additionally, there was a marked deterioration in neurological status (NIHSS 24) [5] and worsening thrombocytopenia, with concomitant worsening of inflammatory markers, onset of fever, and abdominal distension. An abdominal-pelvic CT scan was requested, which revealed splenic ischaemia (Figure 3). The transthoracic echocardiogram (TTE) showed vegetations at the free edge of the mitral valve (Figure 4), which, together with two negative blood cultures after 10 days, Janeway lesions, and the remaining embolic phenomena, made the picture of ME highly suggestive.

**Figure 2:**
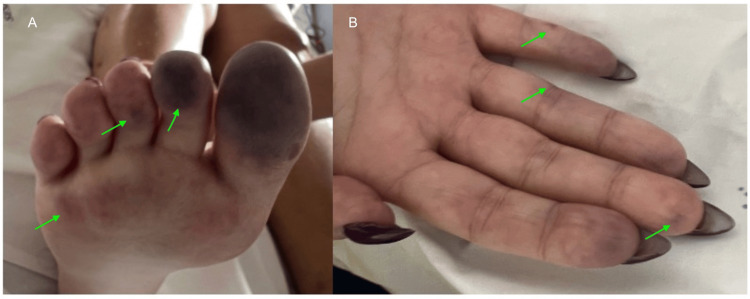
Janeway lesions (A, B) Green arrows point to some of the Janeway lesions scattered across the patient’s hands and feet.

**Figure 3:**
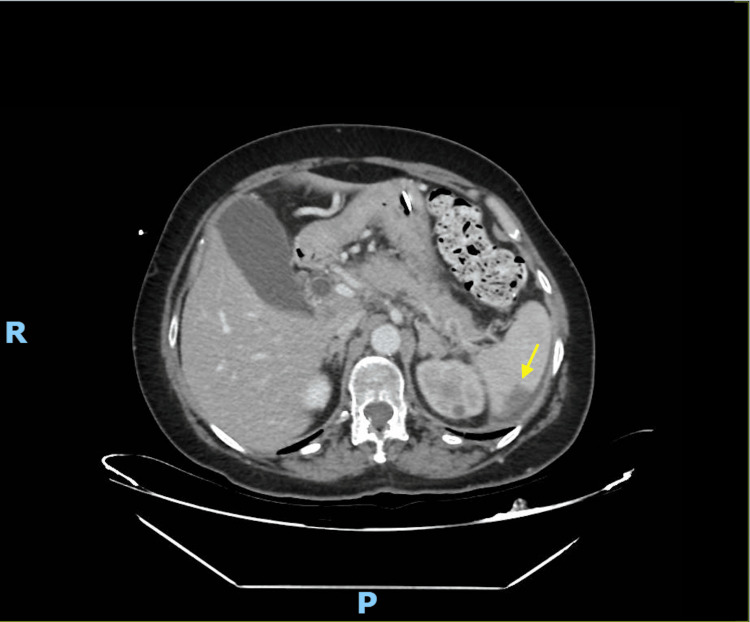
Abdominal and pelvic CT scan showing splenic ischaemia Area of hypodensity in the splenic parenchyma compatible with an ischaemic zone (yellow arrow), measuring approximately 3 × 1.5 cm in its greatest axes on the axial plane.

**Figure 4:**
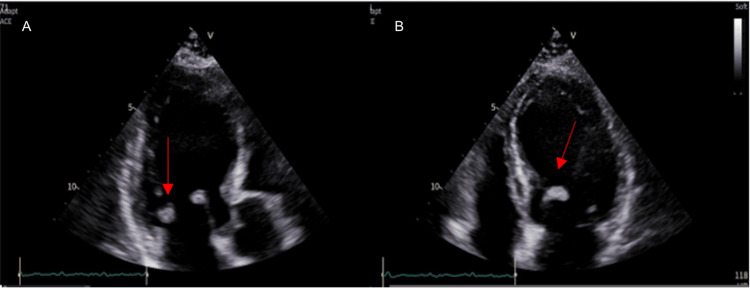
Transthoracic echocardiogram with vegetations at the free edge of the mitral valve A and B show vegetations (red arrows) at the free edge of the mitral valve at two different phases of the cardiac cycle.

Osimertinib was discontinued, as it was suspected to be contributing to thrombocytopenia; however, there was no improvement in the condition. Consequently, given the haemorrhagic risk associated with stroke, it was not possible to initiate therapeutic anticoagulation. In the following days, the patient developed a deep vein thrombosis of the left lower limb and a pulmonary embolism, with progressive clinical deterioration culminating in multiorgan failure and death.

## Discussion

ME is characterised by sterile vegetations composed of fibrin and platelets that attach to the heart valves, predisposing patients to embolisation. It results primarily from a combination of endothelial damage and a profoundly prothrombotic environment, such as that found in neoplasms, particularly adenocarcinomas, as demonstrated in a large-scale study involving autopsies of 50,403 patients, in which 62% of ME associated with neoplasms were due to adenocarcinomas [2]. The exact incidence of ME in the general population remains unknown; it is more common in women (69-75%) and frequently occurs in otherwise healthy valves [1].

Diagnosis is challenging, as the condition can present in a manner similar to IE; therefore, a high degree of clinical suspicion is essential in patients with risk factors for ME, particularly those with neoplasms or autoimmune diseases, who present with a stroke or multifocal thromboembolism [1]. Compared with IE, CNS embolisation in ME is more pronounced. Our patient met the modified Duke criteria for possible IE, with one major criterion and one minor criterion. However, given the presence of lung adenocarcinoma, the various embolic phenomena (including Janeway lesions, splenic, renal, and cerebral ischaemia), and negative blood cultures, the diagnosis of ME was the most likely. The TTE is recommended as the first-line test for diagnosing ME, as it is a cost-effective, safe, and quick procedure. However, it is less sensitive than transesophageal echocardiography (TEE), especially for identifying smaller vegetations, which are common in ME. Therefore, if suspicion is high and the TTE is negative, one should proceed to a TEE [1]. In this case, the vegetation was promptly identified with TTE; thus, no TEE was performed. However, a TEE could have provided us with more details about the vegetation.

The management of ME involves treating the underlying neoplasm and initiating early anticoagulation. The choice of anticoagulation will depend on the underlying condition, the risk of bleeding and the patient’s comorbidities, taking into account the risk-benefit balance [1]. Unfractionated heparin or low-molecular-weight heparin has a favourable profile due to its rapid onset of action, with greater efficacy in cancer-associated thrombosis and more extensive experience in the context of ME [2]. This patient would benefit from anticoagulation to reduce the risk of venous and arterial thrombosis, as well as to treat endocarditis itself.

Evidence suggests that if effective systemic therapy targeting an established therapeutic target is initiated early, it may lead to regression of the neoplasm and, subsequently, to a reduction in thrombotic activity, sometimes resulting in the resolution of the vegetations [2]. Osimertinib is an EGFR inhibitor and is considered a first-line treatment for stage IV EGFR-mutation-positive non-small cell lung cancer, as was the case with this patient prior to the onset of these symptoms. It is known, however, that thrombocytopenia is a recognised side effect of this drug, occurring in 47-51% of cases [6,7]. Disseminated intravascular coagulation could also be considered as a cause of thrombocytopenia, particularly in this complex neoplastic context and in the presence of thrombotic phenomena. However, its management would consist of supportive care and treatment of the underlying cause, which in this case would be advanced cancer [8].

The literature suggests that in patients with platelet counts > 50,000, the risk of bleeding with therapeutic anticoagulation does not increase. However, in the presence of renal dysfunction, as observed in this case, this risk is no longer negligible. Furthermore, the presence of worsening thrombocytopenia during hospitalisation, despite the discontinuation of osimertinib, together with evidence of haemorrhagic transformation of the stroke, made it considerably difficult to initiate standard ME therapy [9]. Another alternative treatment would be cardiac surgery, which is rarely done due to the frailty of these patients, being considered only in specific situations, such as recurrent embolisation despite optimised anticoagulation or refractory heart failure, which did not apply to this patient given the high surgical risk. A palliative approach is often necessary when widespread disease precludes curative or surgical interventions, which was the case here [1,2,4].

The prognosis for these patients is usually poor, particularly in those with advanced cancer, who have a high in-hospital mortality rate. The main predictors of mortality are the presence of splenic or renal infarcts and pulmonary and peripheral embolisms, with the absence of anticoagulation in ME being associated with significantly higher mortality rates. Taking all this into account, our patient certainly had a very poor prognosis, and her outcome was, unfortunately, as expected [1,3]. Figure 5 depicts the timeline of major clinical events.

**Figure 5:**
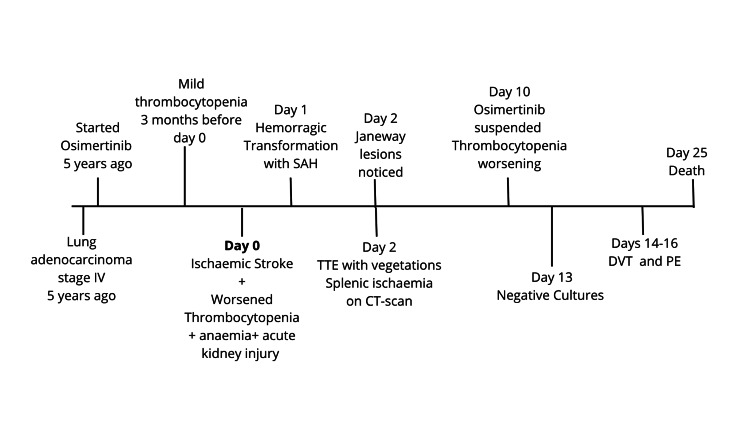
Timeline summarising major clinical events Day 0 corresponds to the admission day. SAH - Subarachnoid Haemorrhage; TTE - Transthoracic Echocardiography; DVT - Deep Vein Thrombosis; PE - Pulmonary Embolism

## Conclusions

This case highlights the importance of a multidisciplinary approach in patients with EM, a condition that affects multiple organs and systems and requires the careful, holistic approach typical of an internist. Furthermore, it presents a complex case in which the therapeutic strategy was limited by severe thrombocytopenia and the haemorrhagic risk associated with stroke, precluding anticoagulation - a cornerstone of treatment - and thereby affecting the patient’s prognosis.
